# Laparoscopic sleeve gastrectomy improves body composition and alleviates insulin resistance in obesity related acanthosis nigricans

**DOI:** 10.1186/s12944-017-0598-z

**Published:** 2017-11-07

**Authors:** Yi Zhang, Cuiling Zhu, Xin Wen, Xingchun Wang, Liang Li, Sharvan Rampersad, Liesheng Lu, Donglei Zhou, Chunhua Qian, Ran Cui, Manna Zhang, Peng Yang, Shen Qu, Le Bu

**Affiliations:** 1 Department of Endocrinology and Metabolism, Shanghai Tenth People’s Hospital, School of Medicine, Tongji University, 301 Middle Yan-chang Road, Shanghai, 200072 China; 2 National Metabolic Management Center, Shanghai, 200072 China; 3 Department of Gastrointestinal Surgery, Shanghai Tenth People’s Hospital, School of Medicine, Tongji University, Shanghai, 200072 China

**Keywords:** Acanthosis nigricans, Obesity, Bariatric surgery, Body composition, Insulin resistance

## Abstract

**Background:**

Acanthosis nigricans (AN) has a close relationship with obesity. It is believed that obesity and AN have the common pathophysiological basis such as hyperinsulinism. This study is aimed to observe the effect of laparoscopic sleeve gastrectomy (LSG) on body composition and insulin resistance in Chinese obese patients with acanthosis nigricans.

**Methods:**

A total of 37 obese patients who underwent LSG in our hospital were selected for analysis. They were divided into simple obesity (OB *n* = 14) and obesity with acanthosis nigricans (AN *n* = 23) group respectively. Body composition was measured by dual-energy X-ray absorptiometry (DEXA). Anthropometric measurements and glucolipid metabolism before and 3 months post LSG were collected for analysis.

**Results:**

Patients with AN got noticeable improvement in skin condition and their AN score was significantly decreased (3.52 ± 0.79 vs. 1.48 ± 0.73, *P* < 0.001).Alleviated insulin resistance and more trunk fat loss than limbs’ were observed in both groups (*P* value < 0.01). In AN group, preoperative android fat mass (FM) was positively correlated with fasting insulin and natural logarithm of HOMA-IR (LNIR) (*r* = 0.622, 0.608, respectively; all *P* < 0.01). Besides, changes in android FM and visceral adipose tissue (VAT) also showed significantly positive correlation with changes in LNIR (*r* = 0.588, *r* = 0.598, respectively; all *P* < 0.01).

**Conclusions:**

LSG had a positive impact on body composition and skin condition in Chinese obese patients with AN. Loss of android FM and VAT might result in the alleviation of insulin resistance in AN patients. Android fat distribution seems to be a potential indicator of postoperative metabolic benefits for obese patients with AN.

## Background

Obesity is regarded as a global pandemic and is defined as being the excessive accumulation of body fat to the point of harming a person’s health, closely associated with a number of chronic diseases such as type 2 diabetes, hypertension, coronary artery disease and cancer [1]. Data from Lancet showed the prevalence of overweight and obesity combined rose by 27.5% for adults and 47.1% for children between 1980 and 2013 worldwide [2]. Compared with countries such as the UK and USA, China had a lower rate of obesity in adults in 2013, but the absolute number of obese people in China is exceeded only by that in the USA. What is more, abdominal obesity appears to be of greater concern than general obesity among Chinese adults. The prevalence of abdominal obesity among Chinese adults, based on WHO suggestions for Chinese, was 37.4% (27.8% in men and 45.9% in women) according to the China Health and Nutrition Surveys in 2009 [3]. Accumulation of adipose tissue in the abdominal region has been associated with insulin resistance (IR), T2DM, and cardiovascular diseases [4–6].

Due to the rising prevalence of obesity, a high prevalence of acanthosis nigricans has been observed recently [7]. More and more patients see a doctor for discolouration rather than obesity, especially in adolescents. Acanthosis nigricans is a mucocutaneous disorder that is characterized by symmetric, skin-colored or brownish, velvety lesions that are especially seen in the axillae, neck, groin, inframammary folds, popliteal fossae, elbows, and umbilical region [8]. Acanthosis nigricans has been associated with many diseases such as type 2 diabetes mellitus, endocrinopathies, malignancies, obesity and drugs. Its relationship with obesity got special concern in the past few years. It is believed that obesity and AN have the common pathophysiological basis such as hyperinsulinism, high level of IGF-1 [9, 10]. For most cases of obesity-associated benign AN, weight loss and lifestyle changes may lead to clinical improvements [11].

In recent years, bariatric surgery has been shown to be superior to nonsurgical approaches for the treatment of obesity in terms of weight loss and remission of type 2 diabetes and metabolic syndrome over both the short term and long term [12, 13]. In the last decade, the number of bariatric surgeries performed worldwide has increased substantially, doubling between 2003 and 2013 from 146,301 to 468,609 procedures per year [14]. Also, SG is currently the most frequently performed procedure in the USA/Canada and in the Asia/Pacific regions as per the IFSO-based world survey. LSG has gained ground because of its good short- and mid-term results, with a lower complication rate than other bariatric surgeries [15–17].

To the best of our knowledge, there was few studies focused on body composition analysis in obese patients with acanthosis nigricans after bariatric surgery. In this study, we aimed to investigate the impact of LSG on body composition measured by dual-energy X-ray absorptiometry (DXA) in obesity patients with acanthosis nigricans over a period of 3 months following this procedure.

## Methods

### Patients

From July 2014 to June 2016, 37 patients (aged 18–65 years) who underwent LSG at the Department of Gastrointestinal Surgery in Shanghai Tenth People’s Hospital of Tongji University were enrolled in this study. The patients were divided into two groups: obesity group (*n* = 14, BMI ≥ 28 kg/m^2^, no acanthosis nigricans, OB) and obesity with acanthosis nigricans group (*n* = 23, BMI ≥28 kg/m^2^, with acanthosis nigricans, AN). Patients included in this study were those with complete data on demographics, weight, and clinical outcomes, and eligible for the 3-month follow-up. All patients provided written informed consent prior to the surgical procedure. This study was approved by the Ethics Committee of the Shanghai Tenth People’s Hospital, Tongji University (no.ChiCTR-OCS-12002381).

Inclusion criteria: Obesity was defined with BMI ≥28 kg/m^2^ according to the guidelines for prevention and control of overweight and obesity in Chinese adults [18]. The following scale for AN was used [19]. Neck severity: 0, Absent or not detectable on close inspection; 1, Present: clearly present on close visual inspection, not visible to the casual observer, extent not measurable; 2, Mild: limited to the base of the skull, does not extend to the lateral margins of the neck (usually < 3 in. in breadth); 3, Moderate: extending to the lateral margins of the neck (posterior border of the sternocleidomastoid, usually 3–6 in.), should not be visible when the participant is viewed from the front; 4, Severe: extending anteriorly (>6 in.), visible when the participant is viewed from the front. Axilla severity: 0, Absent: not detectable on close inspection; 1, Present: clearly present on close visual inspection, not visible to the casual observer, extent not measurable; 2, Mild: localized to the central portion of the axilla, may have gone unnoticed by the participant; 3, Moderate: involving entire axillary fossa, but not visible when the arm is against the participant’s side; 4, Severe: visible from front or back in the unclothed participant when the arm is against the participant’s side. In this study, each subject enrolled with AN had a score greater than 2.

Exclusion criteria included severe hepatic and renal impairments, cardiovascular and cerebrovascular diseases, chronic consumptive diseases such as cancer. Meanwhile, women who were pregnant or lactating were also excluded. Those patients who were clinically verified as having malignant AN were excluded.

### Anthropometric assessment

All measurements were made before and 3 months post LSG. A basic anthropometric examination, including measurements of body weight, height, neck, waist and hip circumference, and assessment of AN, was performed by trained technicians. Participants were weighed barefoot and in light clothing to the nearest 0.1 kg. Height was measured with a fixed wall stadiometer. Neck circumference was measured to 1 mm accuracy with a plastic tape in a standardized manner, horizontally above the cricothyroid cartilage, just below the laryngeal prominence [20]. The waist circumference was measured at the end of gentle expiration midway between the lowest rib and the iliac crest with the patient standing, central obesity as assessed by WC (≥90 cm in male and ≥85 cm in female), while the hip circumference was measured at the greater trochanter [21]. BMI was calculated as weight (kg) divided by squared height (meters).

### Data collection

Glucolipid metabolism indexes, such as fasting plasma glucose (FPG), fasting insulin (FINS), fasting c-peptide (FCP), total cholesterol (TC), triglycerides (TG), low density lipoprotein cholesterol (LDL), high density lipoprotein cholesterol (HDL) were recorded. IR index was determined using the homeostasis model assessment of insulin resistance (HOMA-IR), which was calculated according to the formula: HOMA-IR = FPG × FINS/22.5. The natural logarithm of HOMA-IR (LNIR) was calculated. Dual-energy X-ray absorptiometry (DXA, APEX4.5.0.2, HOLOGIC, USA) was used to measure body composition. Fat distribution was evaluated by the following parameters: fat mass (FM), lean mass (LM), bone mineral content (BMC), and percent fat mass (%FM). In DXA examination, android and gynoid were used to represent two main types of fat distribution. Android mainly referred to body fat around the abdomen. Gynoid referred to body fat around the buttocks and thighs. FM, LM, BMC, and %FM were measured in the whole body and six different regions including arms, legs, trunk, head, android, and gynoid. Body composition consisted of FM, LM, and BMC. The weight of the whole body and each tested body region included the weight of FM, LM, and BMC. Visceral fat analysis was performed by CoreScan™, a software available for the assessment of visceral fat (mass in g, volume in cm^3^ and area in cm^2^) in the android region. Three DXA indexes were calculated: android/gynoid FM (A/G), %FM trunk/%FM legs (trunk/leg), trunk/limb FM (trunk/limb).

### Statistical analysis

Statistical analysis was carried out using SPSS for Windows version 20.0 (IBM, Chicago, IL, USA). All continuous data are presented as the mean ± standard deviation. Independent Student’s t-test was applied in the intergroup comparison, and paired samples t-test was used to test the differences of continuous variables before and after surgery. Correlation analysis was determined using the Pearson coefficient. Differences with *P* (two-tailed) value <0.05 were considered to be statistically significant.

## Results

### Baseline anthropometric and metabolic characteristics of the patients

Table 1 shows the baseline and metabolic characteristics of the patients. At baseline, patients in AN group were younger than those in OB group (29.04 ± 5.85 vs. 42.71 ± 14.13, *P* < 0.001). Compared to OB group, there was no difference in BMI, NC, WC, HC and WHR in AN group. As for glucose metabolism, the levels of FINS and FCP were markedly higher in AN group than those in OB group (*P* = 0.007, *P* = 0.003, respectively), while there was no significant difference in FBG and HbA1c between the two groups. In addition, no significant difference was observed in lipid profile including TC, TG, HDL and LDL between AN and OB groups.

**Table 1:** Clinical and metabolic characteristics of subjects at baseline and 3 months post LSG

Characteristics	OB(*n* = 14)		AN(*n* = 23)	
	0 m	3 m	0 m	3 m
Age(years)	42. 71 ± 14.13	–	29.04 ± 5.85^**^	–
Gender(Male/Female)	6/8	–	12/11	–
AN Score	–	–	3.52 ± 0.79	1.48 ± 0.73^**##**^
BMI(kg/m^2^)	37.79 ± 4.87	30.57 ± 4.53^**##**^	40.56 ± 4.54	31.92 ± 4.21^**##**^
NC(cm)	43.64 ± 3.78	38.50 ± 3.21^**##**^	44.89 ± 4.71	40.59 ± 3.47^**##**^
WC(cm)	119.50 ± 13.93	102.14 ± 12.98^**##**^	120.76 ± 11.07	102.78 ± 11.13^**##**^
HC(cm)	122.07 ± 12.33	107.75 ± 10.23^**##**^	122.43 ± 8.28	109.11 ± 9.13
WHR	0.98 ± 0.07	0.95 ± 0.07	0.99 ± 0.07	0.94 ± 0.07^**##**^
FPG(mmol/L)	7.52 ± 3.23	5.45 ± 1.61^**##**^	5.81 ± 1.62	4.53 ± 0.44^**##**^
FINS(mmol/L)	23.24 ± 8.52	10.68 ± 5.17^**##**^	44.81 ± 33.63^**^	12.53 ± 7.90^**##**^
FCP(mmol/L)	3.49 ± 1.15	2.47 ± 0.65^**##**^	5.18 ± 2.10^**^	2.75 ± 0.83^**##**^
HOMA-IR	8.04 ± 3.73	2.59 ± 1.64^**##**^	12.29 ± 10.94	2.57 ± 1.77^**##**^
LNIR	1.98 ± 0.50	0.79 ± 0.58^**##**^	2.22 ± 0.73	0.76 ± 0.63^**##**^
HbA1c(%)	6.99 ± 1.84	5.79 ± 1.06^**#**^	6.40 ± 1.18	5.21 ± 0.28^**##**^
TC(mmol/L)	4.60 ± 1.17	4.23 ± 0.96	4.58 ± 1.12	4.41 ± 0.79
TG(mmol/L)	1.74 ± 0.66	1.17 ± 0.40^**##**^	1.92 ± 1.62	1.14 ± 0.30^**#**^
HDL(mmol/L)	1.04 ± 0.19	1.10 ± 0.28	0.94 ± 0.23	0.99 ± 0.22
LDL(mmol/L)	2.91 ± 0.97	2.57 ± 0.75^**#**^	2.81 ± 0.86	2.84 ± 0.57

Data presented as means ± SD; ^**#**^ *P* <0.05 baseline vs. 3 months; ^**##**^ *P* <0.01 baseline vs. 3 months; **P <0.01 OB vs. AN group at baseline

### Changes of weight, glucose and lipid metabolism after surgery

As shown in Table 1, skin condition of patients with AN got noticeable improvement and the corresponding AN score was also significantly lower (3.52 ± 0.79 vs. 1.48 ± 0.73, *P* < 0.0001) by 3 months postoperatively. Anthropometric measurements including BMI, NC and WC were significantly decreased in both groups while WHR was reduced notably just in AN group. All glucose indexes (FPG, FINS, FCP, HbA1c) were dramatically decreased at 3 months compared with baseline in the two groups. Moreover, insulin resistance was greatly improved in the two groups. In terms of lipid metabolism, triglyceride levels decreased significantly in OB and AN groups (1.74 ± 0.66 vs. 1.17 ± 0.40, *P* < 0.01; 1.92 ± 1.62 vs. 1.14 ± 0.30, *P* < 0.05, respectively). LDL was significantly lower in OB group. However, TC and HDL levels did not change much in either group (Table 1).

### Changes of body composition

Body composition values in the 3-month period of follow up are listed in Table 2. As shown in Table 2, there was a marked reduction in total and regional body mass and body fat from baseline to 3-month of follow-up in both OB and AN groups (all *P* values <0.01), but not in most total and regional BMC. In particular, the specific analysis of visceral adipose tissues including VAT mass, volume and area showed a pronounced decrease during the entire follow-up period (*P* < 0.01). On the other hand, no significant changes of A/G, trunk/leg ratio were measured throughout the entire period in both groups. In OB group, a significant reduction was observed in trunk/limb ratio (1.36 ± 0.26 and 1.26 ± 0.23, *P* < 0.05).

**Table 2:** Body composition and derived indexes in OB and AN patients at baseline, 3 months post LSG

Variables		OB		AN	
		0 m	3 m	0 m	3 m
Total	FM (kg)	45.78 ± 10.63	36.21 ± 9.05**	47.78 ± 9.02	36.71 ± 8.59**
	LM (kg)	55.29 ± 11.02	46.54 ± 9.67**	62.92 ± 12.12	51.69 ± 9.20**
	BMC (kg)	2.51 ± 0.51	2.51 ± 0.48	2.66 ± 0.50	2.68 ± 0.50
Android	FM (kg)	4.77 ± 1.41	3.43 ± 1.11**	4.82 ± 0.91	3.15 ± 0.96**
	LM (kg)	4.71 ± 0.87	3.53 ± 0.59**	5.03 ± 1.10	3.65 ± 0.81**
Gynoid	FM (kg)	6.29 ± 1.56	5.00 ± 1.21**	6.65 ± 1.52	4.92 ± 1.36**
	LM (kg)	8.66 ± 1.85	6.95 ± 1.36**	10.13 ± 2.03	8.06 ± 1.70**
Trunk	FM (kg)	25.24 ± 6.56	19.20 ± 6.03**	26.47 ± 4.67	18.75 ± 4.75**
	LM (kg)	28.38 ± 4.40	23.16 ± 4.06**	31.16 ± 6.49	24.84 ± 4.72**
	BMC (kg)	0.75 ± 0.13	0.67 ± 0.10*	0.75 ± 0.16	0.72 ± 0.15*
Arms	FM (kg)	6.47 ± 1.85	5.08 ± 0.97**	6.84 ± 1.35	5.50 ± 2.10**
	LM (kg)	5.52 ± 1.75	4.73 ± 1.53**	6.37 ± 1.81	5.36 ± 1.32**
	BMC (kg)	0.33 ± 0.07	0.35 ± 0.11	0.34 ± 0.06	0.35 ± 0.07
Legs	FM (kg)	12.41 ± 3.07	10.44 ± 2.61**	14.29 ± 3.69	10.88 ± 3.36**
	LM (kg)	17.86 ± 4.33	15.13 ± 3.83**	20.96 ± 3.82	17.15 ± 3.51**
	BMC (kg)	0.88 ± 0.21	0.92 ± 0.22*	0.99 ± 0.21	1.00 ± 0.21
Head	FM (kg)	1.66 ± 0.25	1.49 ± 0.25**	1.67 ± 0.27	1.59 ± 0.33
	LM (kg)	4.36 ± 0.57	4.08 ± 0.56*	4.44 ± 0.57	4.34 ± 0.66
	BMC (kg)	0.60 ± 0.14	0.61 ± 0.13	0.59 ± 0.12	0.61 ± 0.12
Vat mass(kg)		1.33 ± 0.28	1.03 ± 0.33**	1.08 ± 0.24	0.72 ± 0.23**
Vat volume(cm3)		1440.45 ± 299.95	1118.45 ± 353.02**	1171.22 ± 254.85	774.06 ± 246.34**
Vat area(cm2)		276.36 ± 57.58	214.64 ± 67.61**	224.72 ± 48.92	148.52 ± 47.21**
Indexes	A/G	1.20 ± 0.14	1.16 ± 0.14	1.26 ± 0.15	1.23 ± 0.15
	trunk/leg	1.18 ± 0.13	1.14 ± 0.13	1.17 ± 0.15	1.09 ± 0.28
	Trunk/limb	1.36 ± 0.26	1.26 ± 0.23*	1.26 ± 0.28	1.13 ± 0.33

Data presented as means ± SD

*P < 0.05 vs. baseline; **P < 0.01 vs. baseline

### Change of fat distribution after LSG in OB and AN group

**Fig. 1:**
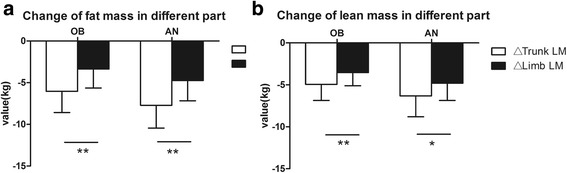
A Comparison of different part in fat mass and lean mass after LSG in AN and OB groups. **a** change of fat mass. **b** change of lean mass. Data are presented as mean. Error bars are SD. *P < 0.05, **P < 0.01

**Fig. 2:**
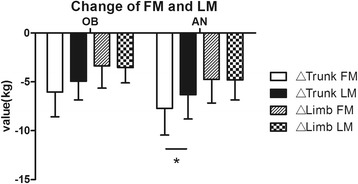
Comparison between the change of fat mass and lean mass in the same body parts after LSG in AN and OB groups. Data are presented as mean. Error bars are SD. *P < 0.05, **P < 0.01

The regional distribution of changes in FM and LM resulting from LSG were also analyzed (Figs. 1 and 2). Overall, the trunk region accounted for more whole-body weight loss than the limbs in both groups. Furthermore, we compared the FM and LM loss of the same body part such as trunk and limbs. The result showed FM reduction was more than LM in the trunk, while reduction of FM and LM in the limbs had no significant difference in AN group. In OB group, FM loss and LM loss occurred at the same time, neither the trunk nor the limbs showed any significant difference between the change of FM and LM in short-time follow up.

### Relationship among FINS, HOMA-IR and body composition

**Fig. 3:**
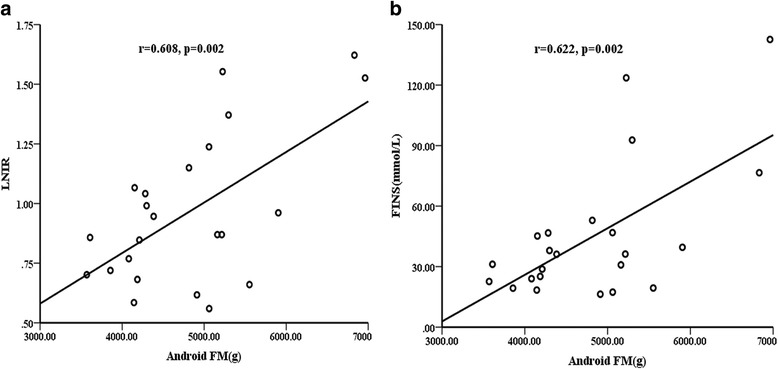
Correlations between android fat mass (FM) and LNIR at baseline in AN group. Android FM was positively associated with LNIR (**a**) and with FINS (**b**)

**Fig. 4:**
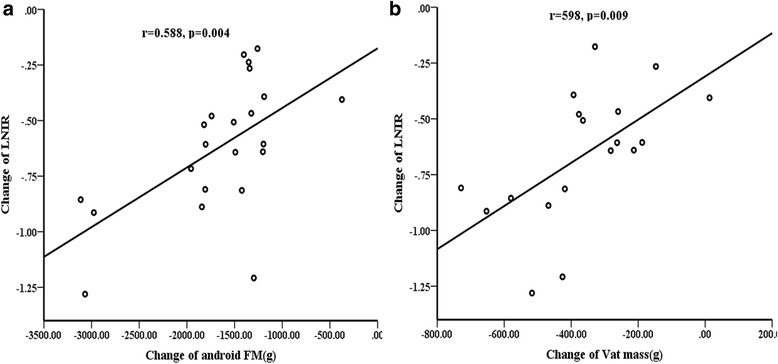
Correlations between changes in LNIR and changes in android fat mass(FM), VAT mass in AN group. LNIR changes were significantly associated with Android FM loss (**a**) and with VAT mass reduction (**b**)

The correlations between insulin resistance and body composition variables were tested in both groups. In AN group, android FM was positively associated with FINS (*r* = 0.622, *P* = 0.002) and with LNIR (*r* = 0.608, *P* = 0.002; Fig. 3) at baseline. Android FM loss was also significantly correlated with LNIR changes (*r* = 0.588, *P* = 0.004; Fig. 4). We further found that LNIR changes were significantly associated with VAT mass reduction (*r* = 0.598, *P* = 0.009; Fig. 4). Oppositely, LNIR was not related to body composition variables, regardless of the baseline or postoperative change in OB group. In addition, no correlations were found between LNIR changes, anthropometric measurements, or clinical variables in two groups.

## Discussion

Acanthosis nigricans was once considered a rare paraneoplastic dermatosis, but is now frequently observed in obese patients, especially in adolescents [22]. The exact pathogenesis of obesity-associated AN is complicated and has yet to be fully elucidated. In the present study, AN patients had obvious higher insulin and c-peptide level compared with OB group. According to the criteria that IR is diagnosed when HOMA-IR is greater than 2.71 [23], patients in the study had severe IR, especially in AN group. Previous most studies also get the same conclusion. In 2002, a study involving a group of total 2205 American Indians showed AN is independently associated with hyperinsulinemia and therefore may be useful as an early indicator of high risk for diabetes [24]. Wiegan et al. [25] observed a sample of 491 obese children and adolescents and concluded that the presence of AN is associated with higher HOMA values. Furthermore, Copeland et al. [26] investigated the association between AN and insulin resistance in children and also observeded that AN was an independent risk factor for the development of insulin resistance. According to the available evidence, IR is regarded as the key mechanism leading to the development of AN in obesity [27, 28]. The most commonly proposed mechanism is the direct and indirect activation of the insulin-like growth factor receptor by hyperinsulinemia, triggering dermal fibroblast, and epidermal keratinocyte proliferation [29]. More confirmatory research is required.

AN is a skin condition that is difficult to treat [30]. Management of obesity-associated AN should treat the underlying cause. If the obesity and the associated IR improve, the cutaneous features of AN can regress [7, 8]. Thus, obesity management is crucial for tackling the problem. Bariatric surgery is gaining favor around the world and thought the most effective treatment modality for obesity and its comorbidities. We further explored body composition and IR in obese patients with AN following bariatric surgery. As expected, in AN group, the skin condition noticeable improved and AN score decreased significantly (3.52 ± 0.79 vs. 1.48 ± 0.73, *p* < 0.0001) over the period of observation. The mean %TWL achieved was 20.58 ± 4.29 and the mean %EWL was 52.68 ± 17.41 at 3 months,which are in accordance with reports by Wang and Vidal [31, 32]. The mean HOMA-IR was decreased sharply and 61% of AN patients had no IR after surgery, confirming the effectiveness of surgery for obesity-associated AN.

In the present study, there was a significant decrease in the total and regional body mass and body fat, suggesting that the body fat accumulation reached a rebalance after LSG. In terms of the postoperative fat distribution, trunk fat mass reduction mainly contributed to weight loss after 3-months. Meanwhile, WC and WHR were significantly decreased (120.76 ± 11.07 vs. 102.78 ± 11.13, 0.99 ± 0.07 vs.0.94 ± 0.07, respectively, all *p* < 0.0001). Since large proportion of abdominal obesity is the characteristics of Chinese obesity and potentially dangerous [33–35], all patients included in our study have central obesity, and the vast majority of them had ratio of trunk/limb fat mass larger than 1.00. Hence, it can be seen that bariatric surgery significantly improves abdominal fat distribution. On the other hand, total and regional lean mass were decreased too. The similar result was observed in obesity without AN patients [36, 37], Robyn A et al. reported the majority of LM loss (18 ± 6% of initial LM) occurring in the first 6 months following surgery [37]. A two-year follow-up after RYGBP showed LM had a moderate decrease at 3 months and stabilized thereafter [38]. Long-term prospective studies about LM loss are necessary.

Furthermore, it is noteworthy that high levels of android FM were positively associated with IR in AN group (Fig. 3). Several studies reported that WC had a strong, liner relationship with IR and could be used in the prediction of metabolic syndrome [32–34]. However, WC or WHR was not significantly correlated with IR in our study, android FM measured by DXA may be more accurate than WC. Change in android FM showed significant positive correlations with change in LNIR(*r* = 0.588, *P* < 0.01), suggesting that the improvement in regional body fat may contribute to the modification of glucose metabolism. Android fat was associated with abdominal subcutaneous and visceral adipose tissue (SAT and VAT). More recently, it has been demonstrated that VAT rather than SAT is the major predictor of adverse events [34–36]. Phillips et al. reported that insulin sensitivity was improved due to continued loss of visceral adipose tissue [38]. Indeed, our study proved that abdominal VAT was significantly reduced after LSG, and LNIR changes were significantly associated with VAT mass reduction (*r* = 0.598, *p* = 0.009), suggesting that abdominal VAT loss after bariatric surgery contribute to the improvement of IR in AN patients. Oppositely, LNIR was not related to body composition variables, regardless of the baseline or postoperative change in OB group. It indicated the post-operation metabolism benefits in obesity and acanthosis nigricans might be different.

There were several limitations in the study. First of all, our study had a small sample size, which potentially affected the accuracy of the analysis. Secondly, the follow-up duration after LSG was short, which caused the long-term effect and safety data for obese patients with AN to be unclear. Third, potential confounders were not properly accounted for due to the study type and the homogeneity of the subjects. Finally, the limited number of patients entering our bariatric surgery program during the period does not allow any conclusion regarding possible gender-related differences in body composition response to surgery. Collection of long-term data is warranted and the effect of gender difference on body composition after surgery should be studied further.

## Conclusion

This study showed a substantial reduction in body composition and obvious improvement in the extent of skin hyperkeratosis and pigmentation in in obese patients with AN followed LSG. Loss of android FM and VAT can result in improvement of insulin resistance in AN patients. Android fat distribution may be a good indicator of postoperative benefits for obese patients with AN.

## Abbreviations

%FM: Percent fat mass
A/G: Android to gynoid ratio in %FM
AN: Acanthosis nigricans
BMC: Bone mineral content
BMI: Body mass index
DXA: Dual-energy X-ray absorptiometry
FCP: Fasting c-peptide
FINS: Fasting insulin
FM: Fat mass
FPG: Fasting plasma glucose
HC: Hip circumference
HDL: High density lipoprotein cholesterol
HOMA-IR: Homeostasis model assessment of insulin resistance
IR: Insulin resistance
LDL: Lowdensity lipoprotein cholesterol
LM: Lean mass
LNIR: Natural logarithm of HOMA-IR
LSG: Laparoscopic sleeve gastrectomy
NC: Neck circumference
SAT: Subcutaneous adipose tissue
T2DM: Type 2 diabetes mellitus
TC: Total cholesterol
TG: Triglycerides
VAT: Visceral adipose tissue
WC: Waist circumference
WHR: Waist to hip ratio
